# A Systematic Review of In Vitro Studies Using Microchip Platforms for Identifying Periodontopathogens from the Red Complex

**DOI:** 10.3390/dj11110245

**Published:** 2023-10-24

**Authors:** Carlos M. Ardila, Gustavo A. Jiménez-Arbeláez, Annie Marcela Vivares-Builes

**Affiliations:** 1 Basic Studies Department, School of Dentistry, Universidad de Antioquia UdeA, Medellín 050010, Colombia; 2 School of Dentistry, Institución Universitaria Visión de Las Américas, Medellín 050031, Colombia; gustavo.jimenez@uam.edu.co (G.A.J.-A.); anny.vivares@uam.edu.co (A.M.V.-B.)

**Keywords:** lab-on-a-chip device, microchip, microfluidics, *Porphyromonas gingivalis*, *Tannerella fosythia*, *Treponema denticola*, periodontal diseases

## Abstract

*Porphyromonas gingivalis*, *Tannerella forsythia*, and *Treponema denticola*, collectively recognized as periodontopathogens within the red complex, have been extensively studied in clinical samples collected from individuals with periodontitis. A lab-on-a-chip (LOC) is a miniature mechanism that integrates various laboratory operations onto a single microchip or a small-scale platform. This systematic review evaluates the application of LOC technology in identifying microorganisms from the red complex. This study adhered to PRISMA recommendations, and the review process encompassed several databases. In the electronic search, a total of 58 reports were found, and ultimately, 10 studies were considered relevant for inclusion. All these studies described effective, rapid, and reliable LOC systems for detecting and amplifying *P. gingivalis*, *T. forsythia*, and *T. denticola*. Compared to traditional methods, the LOC approach demonstrated minimal reagent requirements. Additionally, the results indicated that the amplification process took approximately 2 to 8 min, while detection could be completed in as little as 2 min and 40 s, resulting in a total experimental duration of around 11 min. Integrating miniaturization, speed, accuracy, and automation within microchip platforms makes them promising tools for detecting and amplifying microorganisms associated with the red complex in periodontal diseases.

## 1. Introduction

Periodontitis is an inflammatory infection of the periodontium characterized by periodontal tissue loss. It results from a combination of factors: a susceptible host, the presence of periodontopathogens, and a decrease in or absence of valuable microorganisms for periodontal health. While the configuration of subgingival biofilm varies among individuals, periodontitis progression is associated with a distinct microbiome shift called dysbiosis [1,2]. Several studies have established an association between dysbiosis of the oral microbiome and the development of periodontitis, suggesting a role for the microbiome in its initiation and progression [3,4].

Subgingival plaque collected from periodontal pockets has served as a representative sample for assessing changes in periodontitis-associated microbiota [2,3]. Among these microorganisms, *Porphyromonas gingivalis*, *Tannerella forsythia*, and *Treponema denticola* have been extensively studied in clinical samples [5,6,7]. Due to their frequent co-isolation and strong association with periodontitis, these three species have been categorized as red-complex bacteria, known for their virulence and pathogenicity, triggering chronic inflammation [8,9,10]. Therefore, targeting these bacteria and restoring a balanced bacterial environment in the mouth are critical in managing and preventing periodontal disease [7,10,11,12].

Conventional bacteriological in vitro methods encompass microscopy, cell infection prototypes, and modern molecular, cellular, and immunological analyses [13]. These methods have been invaluable in advancing our understanding of the molecular and cellular aspects of host-bacterial interactions. Nevertheless, they have limitations [14]. Despite improvements in culture techniques, certain microorganisms, like *T. denticola*, remain uncultivable in vitro. Ex vivo and in vitro representations cannot accurately replicate the physiological environment [14,15]. Additionally, molecular tests relying on DNA for microorganism detection may not differentiate between live and dead microorganisms, potentially leading to misleading results, particularly in settings where microbial viability is critical [9,10,11,12]. Subsequently, in vitro and in vivo outcomes may not consistently correspond. Moreover, certain characteristics, like the formation of biofilms, are conventionally examined in simplified settings that do not entirely replicate difficult functional conditions [14,16].

The current progress in lab-on-a-chip technology provides answers to these obstacles and elevates basic bacteriological studies, contributing to the advancement of therapeutic approaches. Lab-on-a-chip represents a small-scale device that combines multiple laboratory procedures onto a particular microprocessor or compact program. Its goal is to efficiently mimic and execute a wide range of laboratory tasks and analyses in a manageable format. In recent years, this knowledge has attracted considerable interest because of its potential uses in healthcare diagnostics, environmental surveillance, and biological investigations [14,17]. This technology entails a reduction in size and the scaling down of various laboratory processes, encompassing sample arrangement, separation, mixing, identification, and evaluation, all onto a compact chip or platform. The integration of these functions into a single device brings several benefits, including decreased sample and chemical quantities, shorter analysis durations, heightened exactitude, and increased mechanization [14,18].

Microfluidic conduits, compartments, regulators, devices, and recognition procedures are commonly produced on a compact chip via processes such as lithography, bonding, and etching. These systems provide exact management and manipulation of liquids, facilitating the manipulation and treatment of small quantities of samples and reagents [14,17,18]. The utilization of microfluidics for microorganism assessment has gained significant traction. These devices, with fluidic channels only a few micrometers in diameter, offer rapid, high-throughput, and cost-effective research options [19].

Some microfluidic devices, such as droplet-based systems, have reported shorter detection times by confining individual bacteria and drugs within small volume plugs, especially for single-drug antimicrobial susceptibility tests [20]. Microchip devices can deliver rapid results, often within minutes, enabling swift pathogen identification in clinical samples. This rapidity is particularly valuable in critical care settings where timely treatment decisions are paramount. Moreover, many microfluidic platforms can conduct multiplexed assays, allowing simultaneous detection of multiple microorganisms or pathogens in a single sample. This capability proves beneficial in cases of co-infections or broad-ranging pathogen screening [17,18,19,20].

An alternative approach to expedite bacterial isolation involves conducting an antibiotic susceptibility test on an inertial microfluidic chip, followed by a method for RNA detection that relies on hybridization [21]. Moreover, following a 3 h incubation, the use of molecular diffusion was applied to identify bacterial proliferation on hydrogel surfaces featuring different gradient areas formed by preparations or mixtures of drugs, resulting in the determination of minimal inhibitory concentration values [22].

It is essential to emphasize the significance of antimicrobial susceptibility testing before prescribing antibiotics, although it is often considered complex and time-consuming. Consequently, point-of-care testing remains a significant challenge. Therefore, lab-on-a-chip technology has the capability to improve the detection of red complex microorganisms in the context of periodontal diseases, offering expedited and personalized diagnostic and therapeutic possibilities for patients. Nevertheless, these aspects have not been comprehensively addressed in a systematic review.

Considering the above, the aim of this systematic review is to assess the application of microchip platforms in identifying microorganisms from the red complex.

## 2. Materials and Methods

### 2.1. Search Approach

The analysis in this study adhered to PRISMA (Preferred Reporting Items for Systematic Reviews and Meta-analyses) criteria [23]. The study methodology involved accessing various databases, including PubMed/MEDLINE, SCOPUS, and SCIELO, and including sources from gray literature. Up until June 2023, MeSH terms and keywords used in searches involved the terms lab-on-a-chip, microfluidics, organ-on-a-chip, microchip platforms, micro physiological systems, bioassays, antimicrobial susceptibility test, biofilms, *P. gingivalis*, *T. forsythia*, *T. denticola*, and studies published in all languages. The subsequent phase involved querying databases by employing Boolean operators (OR, AND) to locate the following terms: “lab-on-a-chip” OR “organ-on-a-chip” OR “microfluidics” OR “micro physiological systems” OR “bioassays” OR “microchip platforms” OR “sensors” OR “biofilms” AND “*P. gingivalis*” AND “*T. forsythia*” AND “*T. denticola*”.

The systematic review procedure was recorded on the Open Science Forum Database under the protocol: osf.io/y52fn.

### 2.2. Selection Criteria

Microfluidic proposals or lab-on-a-chip technology, in conjunction with 3D reproduction and/or bioprinting techniques for organ-on-a-chip technology, should be employed in the in vitro studies incorporated within this systematic review.

We also excluded systematic reviews and meta-analyses, reviews, abstracts, conference papers, concise communications, case reports, patents, and research that did not contain essential information about the manufacturing process.

### 2.3. Question

The current systematic review seeks to address the interrogation: can microchip platforms facilitate the identification of microorganisms belonging to the red complex?

P: experimentation with *P. gingivalis*, *T. forsythia*, and *T. denticola*.
I: microchip platforms.
C: control experiments.
O: identification of microorganisms of the red complex.

### 2.4. Review Course

Two researchers assessed the titles and abstracts of articles to identify those that appeared suitable for inclusion. To address the potential for discrepancies in study selection, a third author (GJ) served as a mediator. The level of agreement among the observers indicated using the statistical test Kappa was determined, and it was found to be significant, with a value exceeding 93.

### 2.5. Compilation of Data

The relevant data from the selected investigations were organized into a table. Every investigator independently compiled the information, and subsequently, a comparison was made. The table encompassed details related to the utilization of the microfluidics methods, containing its essential attributes, such as the constituents utilized in its construction, culture specifics, bacterial strains, and growth conditions, as well as the primary outcomes.

### 2.6. Risk of Bias

The quality evaluation instrument for in vitro research established [24] was employed to evaluate the risk of bias in the investigations comprised in this systematic review. A comprehensive assessment was conducted, considering each study’s objectives, sampling strategy, characteristics of the comparison group, detailed methodological description, operator information, randomization procedures, result measurement, aspects related to result assistance, blinding, and statistical analysis. Based on the scores obtained within this evaluation, each study was assigned an overall quality assessment categorized as low (9–12), medium (5–8), or high (1–4).

## 3. Results

**Figure 1:**
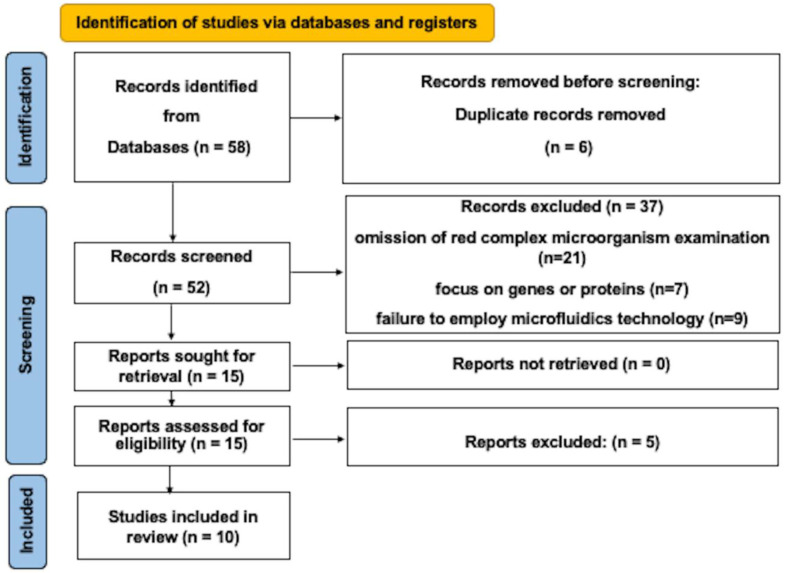
Schema of the selection method.

Out of the 58 reports identified in the electronic search, 37 articles were excluded for various reasons, such as not addressing the examination of red complex microorganisms, focusing solely on genes or proteins, or lacking the use of microfluidics technology. Additionally, six publications that were duplicated were removed. After a thorough examination of the complete texts, five more studies were deemed ineligible based on the selection criteria. Ultimately, this systematic review encompasses 10 papers [25,26,27,28,29,30,31,32,33,34] (Figure 1).

Table 1 depicts the features of the involved reports. The investigations covered were between 2010 [25] and 2023 [30,32].

The studies explored various microfluidic machineries for the detection and amplification of *P. gingivalis*, *T. denticola*, and *T. forsythia*, as detailed in Table 1. All the research endeavors outlined efficient, swift, and reliable approaches for identifying and amplifying these periodontal pathogens. When comparing these systems to conventional methods, it was evident that lab-on-a-chip requires a significantly reduced quantity of chemicals. The outcomes indicated that the magnification of *P. gingivalis*, *T. denticola*, and *T. forsythia* typically took between 2 to 8 min, with detection achievable in 2 min and 40 s, resulting in a total experimental duration of approximately 11 min [26,27,28,29,30]. Additionally, the findings demonstrated that with multi-PCR, the amplification time for shorter amplicons could be reduced to as little as 3 min and 48 s [28]. Moreover, the lowest detectable bacterial concentration was 125 colony-forming units per microliter [26]. Consequently, lab-on-a-chip equipment compromises a substantial advantage in speediness, and the integrated design is highly convenient.

Table 1 displays the various materials and microfabrication techniques employed in the construction of microfluidic systems dedicated to the study of *P. gingivalis*, *T. denticola*, and *T. forsythia*. Silicon wafers with nanoscale chip structures can be produced through photolithography. Lab-on-a-chip often incorporates elements such as reservoirs, chambers, and microchannels. Furthermore, practical elements like valves, mixers, and pumps are designed for the precise manipulation of liquids. As per the findings of this systematic review, polydimethylsiloxane silicone rubber emerges as a widely favored material in laboratory settings for crafting microfluidic devices.

*P. gingivalis* received the highest level of attention among the microorganisms studied here. Nonetheless, most investigations assessed all three microorganisms that constitute the red complex [26,28,29,31,32,33]. Additionally, most of the studies utilized identical bacterial strains for *P. gingivalis* (ATCC 33277), *T. denticola* (ATCC 35405), and *T. forsythia* (ATCC 43037).

All studies exhibited a lack of reporting about certain critical aspects, including sample size calculation, operator calibration, randomization methods, and blinding. Nevertheless, the objectives, inclusion of a control group, experimental methodology, data analyses, and presentation of results were adequately detailed. Consequently, all studies received a score of 6, categorizing them as medium risk of bias based on the assessment tool employed [24]. Moreover, the objectives, conceptual frameworks, outcome variables, and methodologies varied across each model, making it challenging to perform combined quantitative assessments.

## 4. Discussion

Since its inception, PCR has performed a pivotal function in biomolecular diagnostics [35]. PCR finds extensive use in genomic analysis for identifying infectious and hereditary diseases, as well as detecting various bacterial pathogens [26]. During PCR, genes undergo in vitro amplification through distinct temperature profiles for denaturation, annealing, and extension, leading to substantial amplification of targeted sequences [32]. However, conventional PCR thermocyclers have time-consuming temperature variation rates, posing challenges for point-of-care analysis. As a result, there has been significant attention to developing rapid PCR technologies [31,32]. Various PCR technologies have emerged over the years. Notably, an ultrafast photonic PCR approach was introduced, which completed 30 temperature cycles in just 5 min by utilizing plasmonic photothermal light-to-heat conversion through photon-electron-phonon coupling [36]. Moreover, a method known as loop-mediated isothermal amplification was created, necessitating the use of four or more specifically planned primers for the magnification process. While it eliminates the requisite for temperature fluctuations, primer design complexity and the risk of false positives due to cross-reactions or aerosol contamination remain challenges [37,38,39]. A Rayleigh–Bénard PCR cell was also developed, employing natural convection to circulate fluid between temperature zones. Although its design is straightforward, achieving stable natural convection imposes severe needs on the magnitude of the response cavity [40,41,42].

A continuous-flow PCR (CF-PCR) technique was developed, positively amplifying *Neisseria gonorrhoeae*’s DNA gyrase gene in just 18.7 min [43]. This system operates by circulating fluid across indirect canals at varying temperatures. It offers control over PCR product volume through flow rate adjustment, a significant advantage [32]. As outlined in this systematic review, research groups conducted a series of investigations into CF-PCR for rapid pathogen identification [26,27,28,29,30,31,32,33,34]. Significantly, CF-PCR was combined with an electrophoresis microfluidic chip, leading to the development of a single, compact microfluidic device that could detect *T. denticola* within 8 min. Additionally, this device incorporated an automated sample injection system, eliminating the need for external syringe pumps [27]. Multiplex-PCR in serpentine channels enabled simultaneous amplification of *P. gingivalis*, *T. denticola*, and *T. forsythia* [28]. To overcome issues related to cross-reactions, a microfluidic chip incorporating a CF-PCR array was created to amplify all three target genes concurrently. However, the determination still required a separate step of off-line capillary electrophoresis [29]. Ultimately, the concurrent amplification of 12 common focus genes became feasible by guiding the PCR solution into distinct chip areas. This improved the efficiency of CF-PCR and enabled on-site detection, making it suitable for point-of-care diagnostics [32].

Although the benefits of lab on a chip are obvious, this option does have certain drawbacks. Despite the numerous benefits associated with polydimethylsiloxane, several shortcomings have been noted. The capacity to attract small hydrophobic molecules or biomolecules, which might interfere with test outcomes, is its principal limitation in the cellular sector [44]. To elude these actions, methods are being devised to modify the exterior of polydimethylsiloxane [45]. In microfluidic-based research, however, there is an obvious lack of defined protocols [46]. Moreover, the limits of microbial detection that can be attained using microfluidic platforms can significantly differ, depending on various aspects such as the platform, the microorganism under investigation, and the detection approach utilized. Nevertheless, lab-on-a-chip machinery holds the promise of achieving extremely sensitive microbial detection [46,47].

This systematic review has various drawbacks. The available evidence originates from a restricted number of laboratory trials that may have treatment implications. Additionally, these analyses differed in their aims, structures, and approaches, leading to substantial diversity. The included studies also exhibited a moderate level of risk, but they have the potential to identify and enhance the presence of red complex microorganisms.

## 5. Conclusions

Microchip platforms can provide rapid results, allowing for the quick identification of red complex microorganisms within minutes. This speed is especially important in clinical settings where timely diagnosis is crucial for patient management. Many of these devices can achieve high levels of sensitivity, enabling the detection of low concentrations of red complex microorganisms. This is vital for identifying early-stage infections or monitoring disease progression. Moreover, microfluidic assays can be designed to target specific red complex microorganisms, reducing the possibility of false-positive outcomes and improving the accuracy of diagnosis. Developing smaller, portable microfluidic systems for point-of-care applications could enhance their utility in dental clinics and remote areas. Additionally, conducting comprehensive validation studies and clinical trials is essential to establish the reliability and effectiveness of lab-on-a-chip equipment in real-world dental settings.

## Figures and Tables

**Table 1:** Main features of the studies covered.

Purpose of the Microchip	Materials Used	Culture, Bacterial Strains, and Growing Conditions	Main Results	Reference
A method for identifying bacterial cells in deionized water solution, utilizing fluidic electrodes and a hydrodynamic focusing technique is provided.	The chip system includes three-dimensional electrodes and an adjustable gap. A syringe pump is used to precisely control the flows. A computer-programmed syringe pump drives the bacterial suspension and KCl solution from their inlets.	The bacterial culture was compressed to create three flowing layers with varying conductivities on a microfluidic device, using a KCl solution for both sheath flow and fluidic electrodes. The impedance spectra of the bacterial suspensions were measured to determine their sensitivities to concentration changes.	For the current design of the microfluidic chip, the lowest bacterial detection concentration is 10^3^ cells mL^−1^ in deionized water, with quantifiable concentrations ranging from 10^3^ to 10^9^ cells mL^−1^ for *P. gingivalis.* The detection sensitivity of this fluidic electrode system can be modified by adjusting the velocity ratio between the sample suspension and the KCl fluid.	[25]
Using an integrated continuous flow polymerase chain reaction and electrophoresis biochip, a portable all-in-one microfluidic system for rapid pathogen testing has been developed.	The system primarily consists of an innovative pumping unit, two aluminum heaters, and a continuous flow polymerase chain reaction and electrophoresis microfluidic chip. The integrated biochip, made from polycarbonate, was created using plastic injection molding.	Bacterial strains of *P. gingivalis* (ATCC 33277), *T. denticola* (ATCC 35405), and *T. forsythia* (ATCC 43037) were provided by Microbiologics Inc. The polymerase chain reaction solution was pumped into the channel for the reaction at various speeds. Conventional PCR was performed using a T100 thermal cycler.	The results showed that DNA can be amplified in as little as 2′31′′ and PCR products can be detected in as little as 3′43′′, with a minimum quantity of bacteria amplified of 125 cfu per μL.	[26]
Water was used as a substitute for PCR solution, and finite element analysis was used to evaluate the influence of the cross-section, width-to-depth ratio, and length ratio.	The topographical arrangement of the continuous flow polymerase chain reaction chip includes direct and curved microchannels positioned on two heating blocks. The chip was constructed from polycarbonate.	Bacterial strains of *T. denticola* (ATCC 35405) were obtained from Microbiologics Inc. The experiment used 10× TBE buffer, SpeedSTAR HS DNA Polymerase, hydroxyethyl cellulose, and 100 bp DNA ladders.	Conserved sections of bacterial 16S ribosomal DNA were successfully amplified in *T. denticola* within 8 min using the portable continuous-flow polymerase chain reaction chip.	[27]
A continuous flow polymerase chain reaction microfluidic chip was used.	A silicon wafer was spin-deposited with SU-8 photoresist. It was then prebaked at 65 °C for 5 min before being soft baked at 95 °C for 15 min. A photolithography machine carved a pattern onto the photoresist using a photolithography mask.	Bacterial strains of *P. gingivalis* (ATCC 33277), *T. denticola* (ATCC 35405), and *T. forsythia* (ATCC 43037) were obtained from Microbiologics Inc. The sterile paper tip was then placed in a centrifuge tube containing 100 µL phosphate-buffered saline and centrifuged at 10,000 rpm for 10 min. Finally, 2 µL of supernatant was employed as the PCR sample.	The results showed that *P. gingivalis* and *T. denticola* target genes can be amplified in 3′48″; however, *T. forsythia* (641 bp) required at least 8′25′′. When multi-PCR of these bacteria was done, just *P. gingivalis* was identified following 11′20′′. As a result, continuous flow polymerase chain reaction has a significant speed advantage.	[28]
A microfluidic device based on a continuous flow polymerase chain reaction array microfluidic chip was designed and built.	A continuous flow polymerase chain reaction array microfluidic chip, a syringe pump, and two aluminum heating blocks comprised the microfluidic system. The PCR solution was pumped into the microchannels at various speeds via the syringe pump.	The specificity and selectivity of the primers for *P. gingivalis*, *T. denticola*, and *T. forsythia* were determined using a traditional thermal cycler and electrophoresis. (*P. gingivalis* forward: GCGCTCAACGT TCAGCC; reverse: CACGAATTCCGCCTGC; *T. denticola* forward: TAATAC CGAATGTGCTCATTTACAT; reverse: TCAAAGAAGCATTCCCTCT TCTTCTTA; *T. forsythia* forward: GCGTATGTAACCTGCCCGCA; reverse: TGCTTCAGTGTCAGTTATACCT).	The results showed that the shortest amplification time for *P. gingivalis*, *T. denticola*, and *T. forsythia* was 2′07′′, 2′51′′, and 5′32′′, respectively. Moreover, in this microfluidic chip, simultaneous amplification of *P. gingivalis*, *T. denticola*, and *T. forsythia* was achieved in 8′05′′. This research could pave the road for high-throughput DNA amplification, potentially advancing continuous flow polymerase chain reaction technology from the lab to the field.	[29]
A double-layer droplet continuous flow PCR microfluidic device was presented to reduce fluidic resistance.	The integrated droplet continuous flow PCR micro-fluidic chip sketch included a T-junction for droplet generation, a serpentine microchannel for continuous flow PCR, and an outlet reservoir for PCR product detection. The prepolymer polydime-thylsiloxane and curing agent were mixed 10:1 and placed into the duplicate mold.	*P. gingivalis* bacterial strains (ATCC 33277) were obtained from Microbiologics Inc. For PCR amplification, SYBR Green PCR Mastermix was utilized. Sangon Biotech synthesized the primers.	The amplification of *P. gingivalis* was completed in 11′16′′, and the fluorescence signal from the positive droplets was obtained. A microfluidic chip of this type can effectively reduce the high resistance caused by long meandering microchannels and significantly reduce the time required for droplets’ continuous flow PCR.	[30]
An integrated microchip was developed that combines continuous flow PCR with microfluidic chip electrophoresis.	The polydimethylsiloxane prepolymer and curing agent were combined in an equal 10:1 ratio before the polydimethylsiloxane was molded onto the replica mold. The continuous-flow PCR microfluidic chip electrophoresis was made up of two polydimethylsiloxane components.	In the continuous-flow PCR microfluidic chip electrophoresis technique, *P. gingivalis* (TGTAGATGACTGATGGTGAAAACC), *T. denticola* (AAGGCGGTAGAGCCGCTCA), and *T. forsythia* (GCGTATGTAACCTGCCCGCA) were detected. An injector containing the PCR solution was attached to the entrance of the chip’s PCR portion.	The amplification of target genes for *P. gingivalis*, *T. forsythia*, and *T. denticola* was completed in 11 min, and the correct PCR products were recognized in the microfluidic chip.	[31]
The creation of a functioning microfluidic device based on a multiplex circular array-shaped continuous-flow PCR microfluidic chip was discussed.	Soft lithography and plasma oxidation bonding were used to create the microchannels. The layout of the microchannels was initially printed as a mask onto a transparent film. A 10.1 cm silicon wafer was spin-coated with a thick negative photoresist and separately baked at 95 °C for 25 min.	*P. gingivalis* (ATCC 33277), *T. denticola* (ATCC 35405), and *T. forsythia* (ATCC 43037) bacterial strains were received from Microbiologics. Sangon Biotech produced and delivered all the primers.	A system of this type may amplify 12 samples at the same time and detect PCR results on-site using fluorescence intensity. In the microfluidic chip, the volume of PCR reagent required was as low as 5 μL. The PCR reagent took 5.38 min to pass through the serpentine microchannel.	[32]
A disk-shaped microfluidic platform that is chair-side compatible was demonstrated.	The disk-shaped microfluidic platform was created by microthermoforming polycarbonate polymer foils on a chip foundry service at the Hahn-Schickard lab using a hot embossing machine. A polydimethylsiloxane elastomeric mold was heated. The covering polymer foil was also heated, and the air was blasted into it at a particular temperature above the foil’s glass transition, causing it to take on the shape of the mold.	The disk-shaped microfluidic platform contained *P. gingivalis*, *T. forsythia*, *and T. denticola.* The GenEluteTM Bacterial Genomic DNA Kit was used to perform enzymatic lysis on 920 μL of entire saliva, followed by silica column-based extraction. A multiplex real-time qPCR test was used to assess bacterial cell counts using 16S rRNA.	The ability of the disk-shaped microfluidic device to identify *P. gingivalis*, *T. forsythia*, and *T. denticola* in < 3 h was established. When the disk-shaped microfluidic platform was compared to a lab-based reference approach, there was a ~90% agreement between targets recognized as positive and negative.	[33]
It was created in vitro antibodies to *P. gingivalis* for real-time detection of microorganisms in clinical samples. The antibodies were immobilized on a CM-5 sensor chip of a biosensor to detect the presence of *P. gingivalis*.	Anti-*P. gingivalis* antibodies were mounted on a Biacore^®^ 1000 CM 5 sensor chip to study antigen-antibody interactions utilizing the amine coupling approach.	The cells were grown in L-15 media supplemented with 10% fetal bovine serum and incubated at 37 °C overnight. *P. gingivalis* ATCC 33277 was cultivated in a chamber under anaerobic conditions at 37 °C in fastidious anaerobe broth. The lymphocytes were stimulated with heat-killed *P. gingivalis* ATCC 33277 at 10^9^ cfu/mL and incubated at 37 °C.	In surface plasmon resonance, incubation with anti-*P. gingivalis* reduced the bacterial response. The in vitro antibody generation approach established in this study could be employed for effective real-time diagnosis of periodontitis, and the attenuating effects of in vitro antibodies imply a role in passive immunization to prevent periodontitis and its associated risk factors.	[34]

## Data Availability

The data obtained in this review were pooled from the included investigations.
